# CONSORT: when and how to use it

**DOI:** 10.1590/2176-9451.20.3.013-015.ebo

**Published:** 2015

**Authors:** Saulo Gabriel Moreira Falci, Leandro Silva Marques

**Affiliations:** 1 Post-doctorate student (Dental Clinic), Universidade Federal dos Vales do Jequitinhonha e Mucuri (UFVJM), Diamantina, Minas Gerais, Brazil; 2 Adjunct professor of Orthodontics, Universidade Federal dos Vales do Jequitinhonha e Mucuri (UFVJM), Diamantina, Minas Gerais, Brazil

Reconciling scientific research results with clinical practice represents a major challenge to healthcare professionals, including orthodontists.^1^^-^ 4 Current clinical decision-making should be mainly based on clinical trials comparing two or more treatment or diagnosis methods. These trials are known as randomized controlled trials (RCTs) and are considered the gold standard in scientific evidence. The quality of RCTs can be assessed based on criteria pre-established by CONSORT (*Consolidated Standards of Reporting Trials*).^5^

CONSORT is a protocol developed by a group of researchers not only to identify problems arising from conducting RCTs, but also to report, in a full and clear manner, the results yielded by research, thereby facilitating RCTs reading and quality assessment.^5^^,^ 6 ^,^ 7 It comprises a 25-item checklist focused on scientific article writing (available at www.consort-statement.org). This checklist provides us with standards of how the trial was designed, analyzed and interpreted. Thus, it consists in a useful tool that allows the researcher to conduct a RCT and the clinical orthodontist to critically assess the quality of evidence provided.

As a result, the orthodontist is able to employ treatment or diagnosis methods in his clinical practice in a safer and more reliable manner. In addition, he will be able to assess the quality of RCTs throughout its entire structure. In order to render analysis comprehension easier, the CONSORT checklist was divided into six categories, according to the parts of an article: 

**1 - Title and abstract:** the title should be concise and the word "randomized" should be used. The abstract should be structured and include: trial design, methods, main results and conclusions. 

**2 - Introduction: **it should include a brief literature review, the rationale for the trial and the objective or hypothesis, all of which reported in a clear and objective manner.

**3 - Method:** it should be carefully reported as follows: trial design; eligibility criteria for participants, with explanation of rationale for such criteria; how and where data were collected; thorough description of intervention, which allows results to be reproduced; description of sample size calculation; changes during the course of trial, with clear reasons; thorough description of methods used for allocation into the trial groups, participants and evaluators blinding; and proper statistical analysis. 

**4 - Results:** primary intervention results should be assessed for each group; the number of participants, assessment losses and exclusions should also be reported for each group, reasons should be clearly stated; post intervention assessment and follow-up periods should be reported; statistical methods used to obtain values of primary and secondary outcomes for each group (e.g. 95% confidence interval) should be reported. 

**5 - Discussion:** it should present: trial limitations addressing sources of potential bias, imprecision and methodological weaknesses; external validity; applicability and interpretation consistent with results, balancing benefits and harms, considering other published evidence. 

**6 - Other information:** the RCT should be registered and the registry number presented; full trial protocol should be available; sources of funding and other support, as well as the role of funders should be highlighted. 

**Figure 1.**
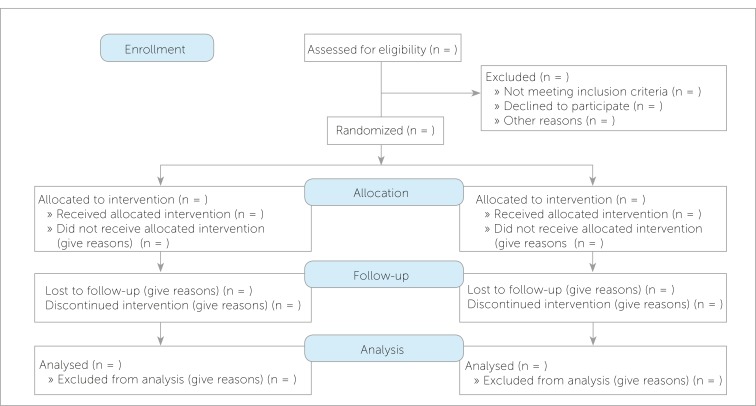
CONSORT flow diagram of the progress through the phases of a parallel randomised trial of two groups (that is, enrolment, intervention allocation, follow-up, and data analysis). Available at: http://www.consort-statement.org/consort-statement/flow-diagram

In addition to the checklist, CONSORT also encompasses a flow diagram which provides the reader with information about how the trial was conducted, reporting enrolment, allocation, follow-up and analysis of patients involved in the RCT (Fig 1).^5^ Importantly, the clinical orthodontist should analyze the presence and quality of this flow diagram in the trial being assessed, since it provides a broad view of how the trial was conducted, in addition to concisely reporting the employed method.

The aforementioned CONSORT criteria have been used for RCTs analysis by more than 600 international periodicals.^8^ In Orthodontics, some prominent journals such as the European Journal of Orthodontics and the American Journal of Orthodontics and Dentofacial Orthopedics began to be based on these criteria in order to accept RCTs for publication. A study reports significant improvements in the quality of RCTs after these journals began to adopt CONSORT criteria. Such progress was particularly noticed in articles published from 2010 on, when CONSORT was revised. Special attention should be given to articles published by the Journal of Orthodontics. Nevertheless, results varied considerably.^9^ On the other hand, another study recently published on the Journal of Evidence-Based Dental Practice concluded that the methodological quality of RCTs in prominent orthodontic journals was below expectations. This study highlighted that its results could be compared to other dental and medical periodicals,^10^ which certainly does not justify low-quality RCTs in Orthodontics.

While critically assessing an RCT, the clinical orthodontist should pay close attention to how coherent CONSORT checklist items and the characteristics of the assessed trial are. Clinicians should understand that, in some studies, there is no need for complete adhesion to all CONSORT items. One example is a study that assessed the level of adhesion to the CONSORT checklist by the American Journal of Orthodontics and Dentofacial Orthopedics. Results revealed that articles conformed with 33 of 37 items from the checklist. According to the author, the following four items were not contemplated: changes to methods (3b), changes to outcomes (6b) after the trial commenced, interim analysis (7b), and trial stopping (14b), which were not rendered necessary for the assessed variables.^8^

Another valuable tool used to guide the clinical orthodontist towards an evidence-based practice is the search for systematic literature reviews and meta-analysis of RCTs.^11^ These have currently been the types of study that provide the best scientific evidence for clinical decision-making. They assess the methodological quality, conduct and writing of various RCTs on the same theme by means of scores, and are assessed in accordance with PRISMA checklist (2009)^12^ which includes the CONSORT checklist items. Thus, RCTs are classified as having low, medium and high risk of bias. However, it is common to find systematic reviews yielding inconclusive results due to low methodological quality of RCTs, which, most of times, results from lack of proper description of what is contemplated by the guidelines. In these cases, the orthodontist should focus on critically assessing RCTs available in the literature, and use the results of trials considered of good methodological quality, so as to guide their practice. 

The orthodontic clinical practice should not be based only on RCTs reading and acceptance of results as being absolute truths. The orthodontist is responsible not only for using such results, but also for critically assessing RCTs. Thus, CONSORT becomes an important tool used to aid clinicians in conducting and assessing the methodological quality of RCTs, which renders these professionals more scientifically aware and confident for choosing the best treatment or diagnosis method to be used.
